# Small-Molecule Modulation of Lipid-Dependent Cellular Processes against Cancer: Fats on the Gunpoint

**DOI:** 10.1155/2018/6437371

**Published:** 2018-08-15

**Authors:** Aswin T. Srivatsav, Manjari Mishra, Shobhna Kapoor

**Affiliations:** Membrane Biophysics and Chemical Biology Lab, Department of Chemistry, Indian Institute of Technology Bombay, Powai 400076, India

## Abstract

Lipid cell membrane composed of various distinct lipids and proteins act as a platform to assemble various signaling complexes regulating innumerous cellular processes which are strongly downregulated or altered in cancer cells emphasizing the still-underestimated critical function of lipid biomolecules in cancer initiation and progression. In this review, we outline the current understanding of how membrane lipids act as signaling hot spots by generating distinct membrane microdomains called rafts to initiate various cellular processes and their modulation in cancer phenotypes. We elucidate tangible drug targets and pathways all amenable to small-molecule perturbation. Ranging from targeting membrane rafts organization/reorganization to rewiring lipid metabolism and lipid sorting in cancer, the work summarized here represents critical intervention points being attempted for lipid-based anticancer therapy and future directions.

## 1. Introduction

Lewis Thomas in the Lives of a Cell [1] underscored the ramifications rendered by the variety of lipids and their structural platforms. Lipid assemblies are noncovalently self-assembling biological constituents that create lipid bilayers, within which lipid molecules can relocate in innumerous ways. Polar lipids, consisting of a hydrophobic and a hydrophilic portion, majorly form the matrix of cellular membranes. The propensity of the hydrophobic moieties to self-associate is entropically driven by water and together with the tendency of the hydrophilic moieties to interact with aqueous environments forms the physical basis of the spontaneous formation of lipid membranes. With advances in lipid-based analytical techniques, lipidomics, we are only beginning to appreciate the astounding diversity of lipids in cells. Eukaryotic cell membranes house a wide repertoire of structural lipids, including glycerophospholipids such as phosphatidylcholine (PC), phosphatidylethanolamine (PE), phosphatidylserine (PS), phosphatidylinositol (PI), and phosphatidic acid (PA) [2]. Sphingolipids constitute another class of structural lipids with ceramide unit as their hydrophobic backbone. The major sphingolipids in mammalian cells are sphingomyelin and glycosphingolipids and sterols represent the major class of nonpolar lipids attributed to their annealed structures that embody a highly condensed hydrophobic area. Various permutations and combinations of the lipid's headgroups and hydrophobic acyl chains add a high degree of complexity to the existing vast pool of known lipids. As much as ~5 % of our genes are devoted to continuously synthesizing and regulating this complex array of lipids, bringing to forefront some exciting questions such as the following: Why is such a complex diversity of lipids required in a cell? Are cells continuously trying to create structural heterogeneity guided by compositional heterogeneity? Is phase coexistence manifested as the existence of domains of coexisting phase(s) functionally relevant? In this lieu, every kind of lipid membrane ranging from eukaryotic to prokaryotic or within the same cell possess unique lipid composition that plays crucial role in not only functional organization but also regulating a plethora of cellular processes. Additionally, steric and electrostatic interactions and hydrophobic mismatch induce distinct domain formation within the bilayer plane providing a platform for organization and assembling of signaling molecules [3–5]. Lipids exist in a multitude of phases each marked by distinct spatial arrangements, molecular structure, and motional freedom of the hydrophobic chains, and being susceptible to environmental conditions like pH, ionic strength, water content, temperature, and pressure is already redefining membrane functionality and offering significant insights to their functional roles in addition to their long held structural roles [6].

Lipids form a considerable part of the dry weight of mammalian cells. A substantial supply of lipids is required for cell proliferation [7]. Usually, during* in vitro* growth of cancer cells, there are abundant nutrients and these cells synthesize fatty acids* de novo*. But, under conditions of stress, the cancer cells usually adapt to the cell growth by scavenging extracellular lipids [8], This scavenging spares the cells the need to supply reducing powers and sources of carbon which are energy demanding. Lysophospholipids are supplied to the lipid pool for growth by K- or H-Ras that stimulate the lipid utilization and uptake. It is well known that mitochondrial fatty acid oxidation produces twice the ATP than that of carbohydrate (glucose) oxidation. Mitochondrial fatty acid oxidation enables cancer cells to survive [9]. Cancer cells acquire a lipogenic phenotype due to high expression levels of the enzyme monoacylglycerol lipase (MAGL). MAGL along with hormone sensitive lipase hydrolyzes triglycerides, stored in adipocytes and other cells to free fatty acids, which now act as a source of energy [10]. Due to this reprogrammed metabolic circuits, various T cells that infiltrate the tumor experience the modulated tumor microenvironment and the altered metabolic network of the growing tumors. Activation of many pathways needs to be accomplished for their function [11]. But the tumor puts several limitations to dampen the T cell activity due to its reprogrammed metabolism like upregulated glucose consumption due to Warburg Effect, as a result the extracellular tumor microenvironment shows reduced glucose levels. Since the T cell for its effective functioning requires energy from its environment in terms of glucose or glutamine [12], due to the tumor metabolism, these sources are rapidly depleted which hence puts the T cell in a dormant state. Therefore, finding ways to modulate these metabolic changes that give cancer cells an advantage to succeed would prove to be useful targets. Some of these have been detailed ahead in this review.

Lipids fulfill many critical requirements in the cell including composing membrane bilayer, storing energy due to their reduced state, acting as first and second messengers in signal transduction, providing functional implementations of membrane-proteins structure and function, and finally recognition processes. Advanced mass-spectroscopy and analytical techniques have allowed sensitive and highly selective analysis of lipids of diverse chemical structures within complex biological samples and testified to their intimate involvement and their aberrations in many diseases such as neurodegenerative disorders, infectious diseases, and cancer. Interestingly, lipidomics have revealed cancer type-specific alteration in the lipidome of cells implying critical roles of lipids in cancer progression and initiation [13–15]. Admittingly, most of the drugs in the market against cancer (also diabetes and inflammation) elicit their effects via binding to their target proteins and regulating the underlying cancer-related cellular process. However in line with the increasing evidence elucidating the role of membrane lipids in regulating numerous cellular functions, they have emerged as attractive molecular targets wherein therapies modulating membrane lipids structures and localization could be developed to control molecular events including changes in cell signaling, membrane protein function, localization, and gene expression related to various pathological states—the so-called “membrane-lipid therapy” [16, 17].

## 2. Membrane-Lipid Microdomains as the Cellular Signaling Hot Spots

High compositional complexity modulates interactions and localizations of lipids in membrane and befittingly influences the diverse phases lipid can form [37]. The same are the authors of spatially constrained distinct regions enriched in certain lipids within the membrane bilayer plane called microdomains [38, 39]. One such microdomain, lipid rafts, is dynamic assemblies of cholesterol and sphingolipids predominantly in the* exoplasmic leaflet *of the lipid bilayer. Underpinning this concept is the propensity,* in vitro*, of the saturated hydrocarbon chains intercalating strongly with cholesterol molecules inducing liquid ordered l_o_ phases [40–42]. The membrane surrounding lipid rafts is fluid-like due to the presence of unsaturated phospholipids forming the liquid disordered l_d_ phase. Thus, the raft domains can be imagined as platforms of l_o_ phases dispersed in the l_d_ matrix of unsaturated glycerolipids. These raft-like assemblies are ordered and tightly packed but are still fluid due to lipid acyl chain packing differences. This, in turn, is governed by the saturation level of the hydrocarbon chains in the rafts compared with the unsaturated state of fatty acids of phospholipids in the l_d_ phase. These rafts act as membrane scaffolds to house various proteins, organize receptors and their downstream molecules, and hence regulate a number of membrane-associated signaling pathways [43, 44]. In this regard, posttranslational modification of proteins such as addition of GPI anchor, acylation, etc. indispensably modulates the membrane affinities of proteins including GPI anchored proteins, epidermal growth factor receptors, estrogen receptors, etc. Membrane rafts are implicated in regulation of cell differentiation proliferation, apoptosis, and necrosis as well as in cancer initiation and progression [45]. Their involvement in numerous tumor models such as colon, prostrate, lung, and breast has been identified [46–49] but their structure, function, and associated signaling pathways are still under intense scrutiny and subject of immense therapeutic interest.

**Figure 1:**
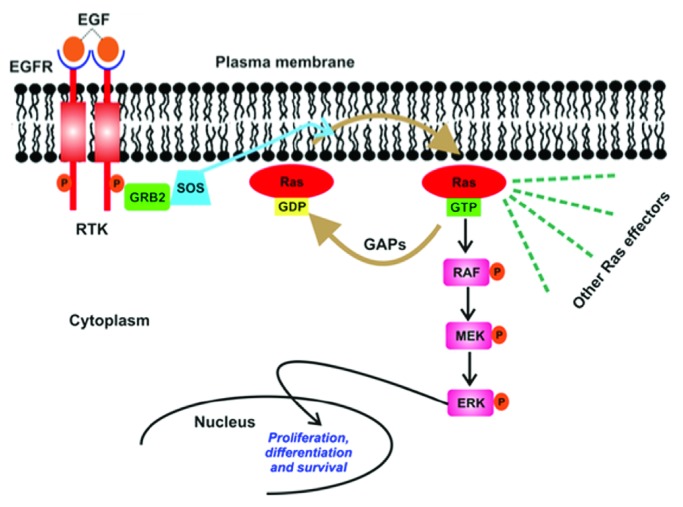
Schematic presentation of MAPK/Ras signaling pathway. Membrane-raft bound-Ras upon activation by variety of extracellular stimuli undergoes a conformational change facilitated by its membrane localization in its switch regions, which is then recognized by other downstream effector proteins in the pathway. This enables signaling events to get amplified downstream producing distinct biological outputs ranging between cell growth, differentiation, apoptosis, and vesicle transport. Any kind of alteration in Ras itself or raft membrane results in various syndromes. Adapted from S. Kapoor, Dissertation TU Dortmund (2012).

On the mechanistic level, a cellular signaling event originates from the raft domains at the membranes with transmission of signals (chemical, biological, and physical in nature) through membrane-bound receptors, e.g., receptor tyrosine kinases (RTKs). Stimulation by the diverse signals promotes receptor dimerization enabling their phosphorylation mostly via their intrinsic tyrosine kinase activity. The resulting phosphorylated residues exposed to the cytoplasm act as docking sites for effector proteins that harbor specialized membrane-loving domains—fostering their recruitment to the membrane. This chain of events induces allosteric changes in the activity and/or affinities on another module of the recruited molecule conveying signals deep into the cell and finally leading to regulation of processes like proliferation, migration, division, and differentiation, among others. Malfunctioning of the cellular signaling both inherent and induced at any given step in space and time results in unchecked downstream cellular functions culminating into various diseases, especially cancer. As an example, we focus on the most fundamental signaling cascade mediating proliferation and differentiation—mitogen-activated protein kinase cascade, MAPK signaling, Figure 1. The pathway gets activated when one of the several extracellular ligands (e.g., epidermal growth factors, EGF) binds to the different plasma membrane-bound receptors, EGFR, leading to the dimerization and autophosphorylation of the cytoplasmic domain of EGFR (i.e., RTK).

EGFR stimulation promotes binding to adaptor proteins GRB2, which further binds Son of Sevenless (SOS) [50]. This binding evokes recruitment of SOS to the plasma membrane, where its close proximity to the membrane-bound small monomeric G-protein, Ras, forms the prerequisite for Ras activation [51, 52]. Ras proteins are membrane-associated molecular switches [53] that lie at the heart of MAPK signaling cascade as signaling convergence modules and hold the place of the first oncogene to be discovered [54, 55] and yet undruggable to date. Activated Ras initiates a series of phosphorylation (and hence activation) events of protein kinases that act as downstream effector proteins of Ras. The most abundant effector protein of Ras is RAF and Ras-activated RAF further activates MEK followed by ERK, which finally travels into the nucleus in its phosphorylated form to activate transcription factors leading to cell proliferation and differentiation [56]. This linear description is rather an oversimplified and abridged depiction of the complex membrane-associated Ras signaling. Mis-regulation of Ras signaling, e.g., by virtue of failed phosphorylation events, improper membrane recruitment of effector proteins, and improper localization with raft domains or mutations account for 50 and 80 % of colon and pancreatic cancers, thus emphasizing the essential role of Ras in normal cell development [57].

## 3. Aberrations of Lipids and Lipid Domains in Cancers: Tangible Targets

Pathological, pharmacological, and nutritional situations strongly regulate lipids in cell with profound biological implications. With advances in high-throughput lipidomics, precise characterization of lipid structures is revealing critical lipid alterations in composition and abundance among various cell types and cancers and surprisingly during varied cellular processes as well [13, 58]. For instance, Eggert et al. [59] demonstrated nice correlation of lipidome changes with cell cycle, with up to eleven different lipid families (chemically distinct structures) accumulating in the dividing cells concluding that cells actively modulate the lipid composition and localization to specific membrane locations required for a particular cellular event. Cellular lipidome remodeling in cancer is manifold and occurs at transcriptomic and lipidomic levels with intriguing complexities [60].

Lipid membranes of cancer cells possess relatively higher negative charge due to increased abundance of PS and PE phospholipids on the exoplasmic membrane surface [61, 62]. On a different note, this contributes to attenuation of repulsion between polar head groups leading to denser lipid packing and concomitantly higher rigidity and poor drug penetration. Thus, exposed PE on the outer membrane of cancer cell represents a suitable molecular target to develop novel cancer therapeutics aimed at specific binding to or selective sorting of PE leading to cancer cell membrane disruption, permeabilization, and finally cell death [63–65]. Cholesterol is another significantly altered molecule within lipid rafts during cancer [66], wherein the levels are strikingly increased compared with normal cells [67]. Higher cholesterol leads to a more rigid and hence less permeable cell membrane [68, 69]. In addition to cholesterol, other phospholipids such as PC and PI are also found in increased abundance in cancer cells. The most foremost effect of elevated cholesterol is higher raft formation and momentous enrichment of specific proteins and receptors such as EGFR, IGF-1, CD44, and CD24 involved in cellular signaling mediating tumor progression and invasion [48, 70, 71]. Thus strategies involved in modulation of lipid rafts are increasingly becoming enticing candidates for cancer therapy [72–74]. Downregulation of ceramide metabolism is another strategy found in cancer cells [75] leading to formation of specialized membrane domains that recruit specific proteins involved in apoptosis highlighting proteins and kinases involved in ceramide metabolism as potential cancer targets. In addition, a wide variety of tumors also show upregulated transcripts involved in lipogenesis and cholesterol synthesis pathway, essential for their development and cancer progression. Lipogenic enzymes such as acetyl-CoA carboxylase and fatty acid synthase display a universal increased expression coupled with specific alterations in lipid messengers (PIs), lipid mediators (leukotrienes), and structural lipids (GSL) in most tumors [76]. In this review we will mainly focus on potential anticancer strategies using small molecules that alter raft assembly, lipid metabolism, regulate lipid sorting, and modulate lipoprotein trafficking inherent in oncogenic signaling. These strategies are presented along with potential targets illustrated with several examples (Figure 4, Table 1).

## 4. Small-Molecule Chemical Biology Tools

Small molecules targeting specific biomolecules and modulating their structure and activity* in vivo* have transformed the field of eukaryotic cell biology. Small-molecule-mediated inhibition of the function of specific proteins has enabled cell biologists to query their functional roles. Most classic example in this regard is of colchicine and paclitaxel as tubulin depolymerizes and stabilizers, respectively, which have provided unprecedented insights into the function of this cytoskeletal protein [18, 19]. Development of a toolbox of small-molecule inhibitors against cytoskeletal proteins and many more has enabled regulation of their structure, function, and localization in such ways that were difficult to achieve solely by genetic approaches. The use of chemical biology tools specifically to study lipid organization offers key advantages. (a) They act fast and their activity can be modulated as a function of dose. (b) They may be reversible or not (covalent binders). (c) They require no manipulation of the chromosome. (d) Inhibitors targeting conserved cellular processes may be applicable across a broad range of species. Due to such salient features, they have a great potential in studying the lipid domain organization in live cells, thus permitting insights into the functional role of membrane organization in cancers and other diseases [20, 77].

## 5. Membrane-Raft Modulating Agents in Cancer

Membrane rafts regulate key signaling molecules and proteins implicated in cancer by modulating their association with and localization with lipid membranes including interactions with other membrane-bound proteins [43, 45, 72, 78, 79]. Thus small-molecule approaches aimed at interrupting the association of such molecules with membrane rafts by interfering with association steps directly or modulating the rafts themselves represent innovative therapeutic ways for prevention and treatment of cancer.

## 6. Small Molecules Acting via Membrane-Raft Disruption

Central roles in the initiation and progression of many tumor types responsible for the alteration of cell cycle, cell adhesion, cell migration, and programmed cell death are regulated by various factors. Lipid rafts and membrane microdomain or compartments play an active role in each of these cell processes by mainly regulating the downstream intracellular signaling pathways [21, 22, 43]. Involvement of cholesterol in maintaining the stability, integrity, and functions aspects of such rafts is indispensable as pharmacological depletion of cholesterol from the membrane causes disruption of membrane rafts and leads to an inappropriate cellular response or function [80–83]. People have reported that rafts play an important role in regulation of cell proliferation, differentiation, apoptosis, and migration; thus modification of their morphology or domains might be intricate in transformation of malignancy, metastasis, and intrusiveness which might get researchers to be involved in studying their structural organization and mechanisms of lipids modulating or depleting agents which causes disorganization of membrane domains and inhibits the tumor progression [43, 83]

**Figure 2:**
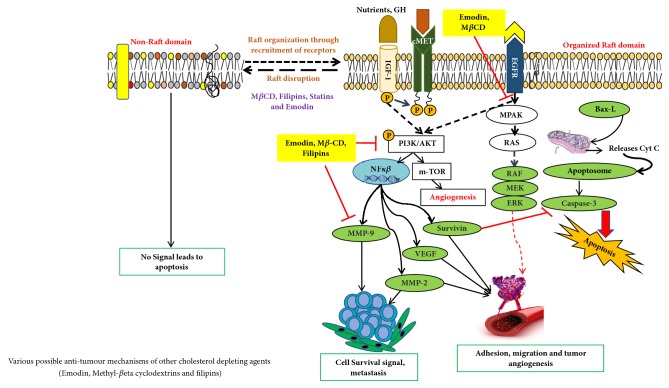
Schematic overview of the targets of action of various cholesterol depleting agents as antitumor drugs.

Membrane rafts are involved in various surface receptor signaling pathways in tumor and lipid depleting agents causing disruption of rafts domains lead to changes in signal response of each [22, 43]. Membrane rafts are involved in EGFR signaling [71], and it has been shown that the activation of this pathway by membrane-raft domains in tumor and cholesterol depleting agents has both kinds of effects on GF receptor-mediated signaling. Several molecules have been shown to cause decreased cancer cell growth, reduced cellular adhesion, and inhibited migration. Among the most promising candidates are flipins, statins, emodin, and methyl-*β*-cyclodextrin-M*β*CD [73] (Figure 2). Emodin (3-methyl-1,6,8-trihydroxyanthraquinon) found in the roots and rhizomes of* Rheum palmatum*, inhibits cancer cell migration by suppressing the PI3K-Cdc42/Rac1 signaling pathway and leads to inhibitory action on cell invasion and cell migration [71, 84]. Some findings dictate emodin suppresses the activator protein Activator Protein-1 (AP-1) and NF-*κ*B (nuclear factor kappa-light-chain-enhancer of activated B cells) signaling [84, 85] pathways, respectively, thus inhibiting the expression of matrix metallopeptidase** 9** (MMP-9) [85] as shown in Figure 2 and hence limiting the invasiveness in various cancer cells. Emodin markedly reduces integrin *β*1 clustering and its colocalization with membrane rafts as judged by cell-based microscopy assays in breast cancer model cell lines [86]. Furthermore, its mechanism of action includes suppression of translocation of integrin *β*1, and focal adhesion complex (FAC) from cytoplasm to membrane rafts, mainly attributed to reduced cholesterol levels in the membranes, thus hampering efficient raft-signaling-platform formation [85]. Thus, the inhibition of membrane-raft clustering or activation of raft disruption by emodin is the underlying mechanism leading to suppression of integrin clustering and FAC formation, and hence halting oncogenic signaling signalling dependent on integrin which was confirmed through confocal analysis; the above analysis approves emodin as a favourable candidate for the novel therapeutic agent in the treatment of cancer metastasis [86] as shown in Figure 2. Methyl-*β*-cyclodextrin (M*β*CD), a water-soluble polymer and a cyclic oligosaccharide, absorbs cholesterol from the cell membrane and has been shown to impair actin polymerization, cell migration, Akt (also known as protein kinase B, PKB) phosphorylation, protein kinase C translocation, and EGF-induced cell adhesion in selective cancer cell models [23, 87, 88]. Interestingly, due to the biochemical effects of M*β*CD, this molecule has been chemically modified to serve as platform for cellular lipid shuffling enabling generation of asymmetric model membrane systems [89–91]. The major limitations of using M*β*CD is an acute process due to comparatively short term of treatment [92]. Thus, statins are more physiological and a practical approach underscored by the fact that chronic cholesterol depletion is better in targeting cancer [92, 93].

**Figure 3:**
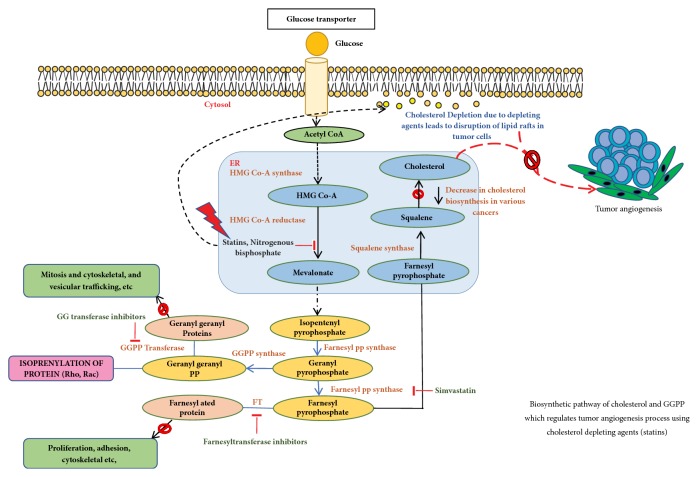
Schematic representation of the biosynthetic pathway of cholesterol and GGPP which regulates tumor angiogenesis process using cholesterol depleting agents (statins).

Statins, the first committed inhibitors of the mevalonate pathway, act at an early step in the synthesis of cholesterol [94]. Statins are the best selling drugs in clinical history [24], and they are used to treat hypercholesterolaemia and dyslipidaemia. They reversibly inhibit the HMG-CoA reductase enzyme function, the rate limiting step in cholesterol biosynthetic pathway. Statins also inhibit the isoprenoids production such as farnesyl pyrophosphate (FP) and geranylgeranylpyrophosphate (GGPP) [94] as shown in Figure 3; they activate the cellular signals by releasing or expressing various proteins with GGPP (RhoA and Rac1) and FP(Ras) lipid modifications. The primary mechanism of HMG-CoA reductase enzyme is to convert HMG-CoA into L-mevalonate and coenzyme A [95], Figure 3. Statins by inhibiting HMG Co A reductase activity reduce cholesterol concentration and also control the expression of LDL receptor [85]. They also reduce the synthesis of Apolipoprotein B100, hence inhibiting the secretion and synthesis of triglycerides. The statin family consists of several drugs—synthetically derived (fluvastatin, cerivastatin, atorvastatin, rosuvastatin, and pitavastatin) and naturally isolated from fungi (lovastatin, pravastatin, and simvastatin)—that are notoriously known for inhibiting oncogenic signaling, inhibiting cell invasion and metastasis in cancer cells through disintegration of membrane rafts resulting from reduced cholesterol levels [25, 95]. Rosuvastatin inhibits prostate cancer cell growth and inhibits angiogenesis [26] and Simvastatin, another drug belonging to the statin family, acts by downregulating PI3K/Akt/Caspase-3 signaling and Fas translocation mainly by modulation of raft assembly [96]. Furthermore, simvastatin blocks Ras-membrane localization and downmodulates H-Ras protein at the posttranslational level [27]. It also selectively dissociated latent membrane protein 1 (LMP1) from membrane rafts and reduces activation of NK-*κ*B signaling culminating to apoptosis [28, 29] and helps in survival of severe combined immunodeficiency (SCID) mice with lymphonomas [29]. Lipid rafts with its arsenal of lipid and protein components involved in many signaling pathways increase the possibility of potential targets for cancer treatments. On a different note, artificial membrane models serve as a promising target in the treatment of various mechanisms involved in cancers, i.e., decrease in cell adhesion and inhibition of cell proliferation, motility, and tumor progression.

Recent single molecule tracking techniques have elucidated that actin-based cytoskeleton structures on the cytoplasmic surface of the plasma membrane are also a key player in inducing membrane domain organization or partitioning into small compartments underpinning the cellular dynamics of protein and lipid lateral diffusion. Such a model implicit in the picket fence model states that the membrane-actin skeleton interactions induce temporary confinement of transmembrane proteins thus generating transient domains that function as signaling host spots similar to rafts. Many receptors like the G-protein coupled receptor (GPCR), transferrin receptors, etc. have been assigned to such transient domains with the membrane skeleton mesh and are also enriched with cholesterol and sphingolipids [97–100]. Specifically, cancer cells having the ability to metastasize depend on this machinery for invasion of various tissues—both local and at distant sites with differential dynamic reorganization of actin [101]. Actin polymerization at the plasma membrane causes protrusion in the cell that dictates the direction of the migration and membrane-lipid raft proteins at focal adhesion points help in detachment from the extracellular matrix. Prostate cancer cells are dependent on Src, focal adhesion kinase (FAK), Cav-1, cavin-1, and actin cytoskeleton for facilitating their adhesion or detachment from extracellular components [102]. A study in 2010 showed that treatment of mice with Cav-1 antisera reduced the development and growth of primary site tumors and metastasis [30], as such targeted activity on proteins that work along with actin-based protein kinases like FAK is achievable using small molecules, e.g., emodin. Along similar lines targeting microtubules that are key components of the cytoskeleton essential for development and maintenance of cell shape, cellular signaling, division, and mitosis is also a promising anticancer strategy. Microtubule poisons like Vinblastine and Vincristine are already in clinical use against cancers like testicular cancer and leukemia [32, 103]. The interplay of actin cytoskeleton with lipid diffusion and interactions with membrane rafts and proteins is known to contribute to cellular migration and growth and immune cell activation, thus highlighting the prospect of targeting lipid/membrane-cytoskeletal interactions for disrupting the ensuing downstream oncogenic signaling.

## 7. Small Molecules Acting by Stabilizing Membrane Domains Involved in Apoptotic Signaling

**Figure 4:**
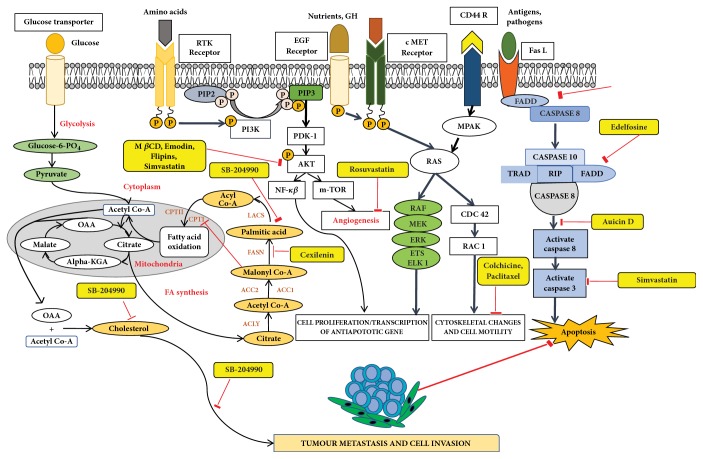
Schematic overview of targeting lipid associated cellular functions in cancer with small molecules. All inhibitors are highlighted in yellow for clarification.

Recent studies have elucidated membrane rafts to form signaling platforms capable of activating pro- and antiapoptotic pathways susceptible to pharmacological perturbations aimed at stabilizing these special apoptotic-linked raft domains [31]. Activation of proapoptotic pathways begins with activation of proapoptotic membrane receptor molecules via oligomerization by agents that promote raft integrity in the absence of receptor ligands. There are two major apoptotic pathways, extrinsic and intrinsic, that originate from membrane rafts [104]. The extrinsic pathway is kick-started by death receptors, e.g., Fas. Following stimulation by its ligand-FasL, Fas undergoes clustering and recruits adaptor protein, Fas-associated death domain-containing protein, FADD [105]. FADD interacts with procaspase-8 forming the so-called death-inducing signaling complex (DISC) that leads to activation of downstream signaling and eventually apoptosis [106]. Activation or clustering of Fas receptor or death receptors in general is critically dependent on membrane rafts to trigger apoptotic signal transduction and is amenable to small-molecule perturbations as follows (Figure 4) [107]. Resveratrol is shown to induce apoptosis in colon cancer cells by redistributing Fas among other death rectors in membrane rafts [108]. Avicin D, a plant triterpenoid, selectively inhibits growth of tumor cells via activation of caspase pathway, i.e., regulated Fas translocation into membrane rafts and subsequently interactions with FADD and pro-caspase 8 to form DISC and hence cause cell apoptosis (Figure 4) [109]. Finally, along similar lines, Edelfosine (1-O-octadecyl-2-O-methyl-rac-glycero-3-phosphocholine), a synthetic lipid, induces apoptotic response by accumulating in the membrane rafts and altering their lipid-protein concentrations and organization [105, 110–112]. This leads to coclustering of FADD and pro-caspase 8 into membrane rafts and thus activated formation of DISC. Remarkably edelfosine is highly selective for leukemia cells and solid tumors compared with normal cells, where it targets only the plasma membrane rafts of leukemia cells and endoplasmic rafts of solid tumor cells [113].

## 8. Small Molecules Rewiring Lipid Metabolism in Cancer

Cancer cells display a highly distinct metabolic growth profile compared with nontransformed normal cells. The metabolic reprogramming of the enzymes of various pathways of cell growth forms the underlying basis of cancer. One of the most implicated pathways that are heavily tinkered within cancer is lipid metabolism. Lipid metabolism is linked closely with the glycolytic pathway by virtue of it providing the required starting substrate—acetyl-CoA—for fatty acid (FA) synthesis. Lipids play key roles in this network, as they are crucial for the formation of cell membranes and also act as signaling messengers. Due to the enormous upregulated growth rate of cancer cells, relatively larger amounts of lipids are required to keep up with alarming rates of growth, proliferation, energy storage, and production of signaling molecules [76, 114]. Targeting lipid metabolism, which encompasses perturbing synthesis, oxidation, and mobilization of lipids, is a promising strategy in cancer treatment. One of the important steps in lipid metabolism is the formation of fatty acid, which uses acetyl-CoA as a substrate. Acetyl-CoA is either obtained from the glycolytic pathway via the conversion of pyruvate or obtained by the breakdown of citrate into acetyl-CoA and oxaloacetate by cytoplasmic ATP citrate lyase (ACLY). Acetyl-CoA binds with malonyl CoA (formed via the carboxylation of acetyl-CoA) to form palmitate, which is a starting product of FA synthesis via the enzyme fatty acid synthase (FASN). Inhibitors against ACLY will lead to the reduced production of acetyl-CoA and in turn reduce the levels of FAs that are formed. ACLY inhibition has been shown to cause growth suppression and induce apoptosis [115, 116]. SB-204990 is shown to inhibit ACLY and therefore block the synthesis of FA and cholesterol (Figure 4). This causes a block in the cancer cell growth and the suppression of tumor, leading to cell death [117]. The next main step amenable to small-molecule targeting is the formation of palmitate by FASN. Palmitate is then converted by a set of enzymes to form an array of saturated and unsaturated FAs. FASN has been well documented with regard to its role played in cancer and is exploited extensively as anticancer target [118]. As most normal cells prefer exogenous sources of FAs, targeting FASN has been demonstrated to be a viable approach as it reduces the de novo FA synthesis in cancer cells. For example, cerulenin, an antifungal agent, is one such inhibitor of FA synthesis, which reduces FA synthesis and rescues tumorous cells [119, 120]. Another such drug is C75 that has been shown to cause the inhibition of FASN [121].

## 9. Small Molecules Targeting Lipid Relocalization and Lipoprotein Sorting

Next to targeting the enzymes involved in lipid biosynthesis, targeting lipid oxidation and mobilization/localization are fruitful therapeutic avenues gaining recent interest. Carnitine palmitoyl transferase 1 (CPT1) is an enzyme involved in the *β*-oxidation of FAs, where it facilitates the movement of FA-CoA from the cytosol to the mitochondria across the mitochondrial membrane. Etomoxir and perhexiline are two small molecules shown to be effective against tumors and curb their proliferation via targeting FA oxidization mediated through CPT1 (Figure 4) [122, 123]. The FAs once successfully translocated can either be diverted for storage or be mobilized from stores as and when needed. The enzymes involved in these mechanisms have proven to be suitable targets for cancer therapies. Glycerol-3-phosphate acyl transferases (GPATs) and its isoforms enable formation of diacylglycerols (DAG) and triacylglycerols (TAG), which are then directed towards storage, while enzymes like monoacylglycerol lipase (MAGL) mobilize FAs from their reservoirs. CT-30501 inhibits GPATs while JZL184 inhibits MAGL. These small molecules help in suppression of tumor growth and induce apoptosis, respectively [124]. The above are just a few examples of the use of small molecules to target lipid metabolism and associated processes in cancer cells and form a firm foundation of lipid-targeted cancer therapies. One of the salient features of lipid membranes is the asymmetric distribution of lipids—aminophospholipids phosphatidylserine (PS) and phosphatidylethanolamine (PE). They are largely present in the leaflet facing towards the cytosol; however upon flipping on the extracellular cell surface they act as markers for signaling pathways. Therefore, this feature of the membrane's asymmetric distribution of composing lipids makes it an important target to fight against human pathology, and certain lipids are extensively used as biomarkers against cancer attributed to the fact of cancer cells expressing high levels of PE and PS exposed on its outer leaflet. Various factors have been reported for the loss of asymmetry in plasma membrane like transcriptional activation due to increase in calcium concentration, inhibition of APTLs, oxidative stress, and transient hypoxia in tumor cells which activates sphingomyelinases. Activated sphingomyelinases eventually leads to disintegration into ceramide and reduces the stability of bilayer leading to membrane blebbing. This in turn activates the proapoptotic signaling pathways, which redistributes the PS to the outer leaflet of the membrane. In recent reports, the loss of asymmetry is mainly due to the reduction of translocase and activation of scramblase enzyme [125]; thus exposure of PS to the outer surface serves as an apoptotic marker in all the cells [125, 126], and this might also potentiate the activity in macrophages for killing the tumor cells. PE acts as a structural component of cell wall as well and is implicated in many cellular processes like cell division and cell death; thus a highly sought-after anticancer target [2]. Recently, SapCDOPS nanovesicles were used to detect PS on the outer surface of the tumor cells and also targeted to induce cell death both* in vitro* and* in vivo* [127]. Cyclotides are cyclic peptides that have a high affinity to target and bind to PE head groups modulating their localization and disturbing downstream cellular functions involving PE. Along with cyclotides, there are two lantibiotic peptides—cinnamycin and duramycin—that are also PE specific and are produced by Gram-positive bacteria [126, 128]. The binding of both these types of peptides has a membrane disruption effect that causes cell death and proved effective in imaging subcutaneous tumors; these findings indicate that externalized PE may be a general maker of tumor vasculature [129, 130].

Lipids, apart from being intimately involved in cellular functions and cellular signaling as isolated modules, add another level of complexity by their covalent attachment to proteins—posttranslational protein lipidation—that forms the heart of membrane-associated signaling in cells, e.g., small GTPases, such as Ras, Raf, and ARFs. A classic example of addressing oncogenic signaling involving lipidated proteins is via targeting the protein lipidation leading to improper membrane-raft localization of these proteins causing nonfunctional signaling platforms and hence subdued oncogenic signaling. This aspect is greatly exemplified by the class of lapidated protein-Ras. The lipid moieties attached to the protein consist of a palmitoyl group and 1-2 farnesyl lipid anchors. Ras was the first oncogene to be discovered and is involved in many human cancers; however small-molecule targeting of Ras still remains an unmet task in cancer therapy. In 1989, one of the first drugs to be thought to target Ras was lovostatin. Farnesyl pyrophosphate farnesylates Ras and it is a key intermediate of the mevalonate-cholesterol biosynthetic pathway. Being a HMG-CoA reductase inhibitor, lovostatin was shown to block the mevalonate-cholesterol biosynthetic pathway and hence the farnesylation lipidation of Ras. However, requirement of much higher dosage for selectively blocking farnesylation leads to adverse effect on cholesterol biosynthesis and unspecific cell death, thus making the journey of lovastatin quite short-lived regarding clinical targeting of Ras Farnesylation [131]. This failure paved the way for the discovery of the enzyme involved in the farnesylation of the Ras proteins. In 1990, farnesyltransferase (FTase) enzyme was isolated and characterized [132]. One of the attempted ways by which membrane-associated Ras oncogenic signaling has been targeted is via inhibiting the activity of farnesyl transerfases to block farnesylation of Ras and hence reduce its membrane-raft association and concomitant signaling in cancer cells [133–135]. More than two decades have been invested to exploit this approach as a practical anticancer therapy, but it has still met with many deadlocks mainly attributed to the nonselective nature of farnesyltransferase inhibitors [136]. Specifically, the key reason for this failure in clinical trials is the presence of an alternate lipidation pathway, i.e., the compensatory activity of geranylgeranyltransferase-I that modifies Ras with geranylgeranyl instead of a farnesyl group upon treatment with farnesyltransferase inhibitors. This still leads to proper Ras localization and hence unaltered oncogenic signaling and cancer.

However, amidst such failed attempts, recently Waldmann and coworkers have demonstrated an exciting alternative to target Ras-associated cancer by mislocalizing Ras lipoprotein not channelized via blocking the lipid attachment but by an innovative chemical biology approach [137]. Lipidated Ras is trafficked through a prenyl-binding protein, PDE*δ*, in cells that sustains the spatial orientation of the Ras superfamily of proteins [138]. Recently high specificity of PDE*δ* towards K-Ras trafficking to reach plasma membrane rafts to initiate signaling was demonstrated [139, 140], and the same was exploited by designing small molecules such as Deltarasin (Figure 4) and related analogs to block the binding pocket of PDE*δ* leading to K-Ras mislocalization and downregulated cancer signaling leading to reducing cell proliferation and finally cancer cell death [137, 141]. Along similar lines, Salirasib, a small-molecule housing a farnesyl moiety, competes with Ras for binding to Galectins, the Ras escort binding protein that contains a complementary farnesyl binding site [36]. This leads to Ras mislocalization and halt of oncogenic signaling as observed with Deltarasin. These studies provide a proof-of-concept platform and opens various channels aimed at targeting lipid-mediated cellular functions in unprecedented ways.

Although a lot of work has to be yet done in identifying membrane specific small molecule against cancer, the effects of presently available small molecule on the membrane specific organization and signaling are proven as effective against the malady* in vitro.*

## 10. Conclusions and Future Directions

The quest for targeting cancer using varied chemical and genetic approaches still is faced with enormous hurdles and generates an unmet need to develop therapeutic approaches inspired by careful inspection of modulated cancer cell attributes. One of the aspects gaining considerable attention recently has been the altered lipid repertoire of cancer cells leading to modulated membrane-dependent cellular processes including membrane organization and cellular signaling, strongly contributing to tumor growth and metastasis and understanding the underlying mechanism behind the same to elucidate potentially novel targets and pathways against cancer. In this review we focused on some of the most promising lipid associated candidates and processes for anticancer targeting by small molecules. Ranging from targeting of lipid enzymes involved in the lipid metabolic pathway to the proteins and lipids that help in lipid organization, oncogenic lipoprotein sorting and signaling membrane micro-domains-rafts have proven to be highly crucial to not only contemplate their therapeutic aspect but also address and unveil specific mechanisms of lipid deregulation in cancer. The various small-molecule-based drugs and tool compounds that have been discussed so far have shown promising results in targeting lipid-dependent processes in cancer cells. Drugs like Salirasib that inhibit Ras function have shown the ability to act in multiple ways and have been used in Phase I clinical trials showing good results with the drug being well tolerated. Deltarasin which targets K-Ras downstream signaling and K-Ras localization may have potential to achieve better efficacy in the long run. Although Statins look the most prospective in cancer treatment, there are side effects to their use. In addition, they all act predominantly by remodeling membrane rafts composition and organization but lead to distinct downstream effects on the oncogenic signaling in various cancer models. This brings to the forefront membrane rafts as “selective cancer therapeutic targets,” with the structure, function, and associated raft-signaling pathways being subject of extensive studies. As such, a better understanding of the structural, conformational, and functions aspects of raft biology would foster exploitation of membrane rafts for developing personalized cancer therapy for targeting distinct raft-associated oncogenic signaling in various cancers. In this regard, recent surge in technological advancements in super resolution microscopy (SRM) is already providing invaluable information on the distribution and organization and dynamics of plasma membrane components to bendings [142] that occur during clathrin mediated endocytosis [143]. Recent advancements in the field of technology hold promising scope of improving the resolution and sensitivity of point localization in SRM based methods [144].

Further chemical biology investigations on the regulation of membrane-lipid-dependent signaling pathways in cancer cell may provide novel targets for therapy and elucidating the role of distinct lipid signaling molecules will offer innovative therapeutic opportunities for development of anticancer drugs. However, discovery of lipid-dependent novel targets and novel signaling pathways in cancer biology strongly lies at the hand of discovering innovative small molecules. Generally, small molecules with well-defined targets permit obtaining novel insights into biological processes and extensive analysis of their structure-activity relationships permits chemical modification for improving efficacy of such candidates. Though nature is a comprehensive source of molecules with a variety of bioactivities, in recent years, a burst in organic synthesis strategies and synthesis of organic molecules generated via innovative hypothesis generating platforms, such as diversity oriented synthesis (DOS) biology oriented synthesis (BIOS), and activity oriented synthesis, have enriched the pool of small molecules in an informed manner, thus promoting their use as research tools to explore previously uncharacterized biology and elucidate novel targets for drug discovery. Given the limited number of drug targets addressed till today in cancer and immunology, including protein kinases, G-protein coupled receptors, and ion channels, development of new small organic molecules rightly fits the criteria for meeting the ever-increasing need for new therapeutic targets. Finally, identification of the molecular targets of such new compounds still remains a major bottleneck, underscoring the demand for appropriate methodologies to elucidate the targets of small molecules in a relatively unbiased and timely fashion.

## Figures and Tables

**Table 1:** Small molecule inhibitors targeting lipid-related cellular pathways for cancer treatment.

Small Molecule	Target or mechanism of action	Ref.
(1) Emodin 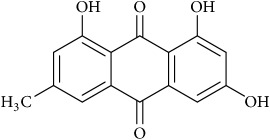	(i) Suppression of PI3K-Cdc42/Rac1 signaling pathway (ii) Hampers efficient raft signaling-platform formation	[18–20]

(2) Rosuvastatin 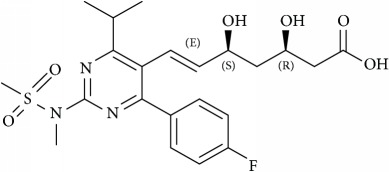	(i) Modulate raft assembly	[21]

(3) Simvastatin 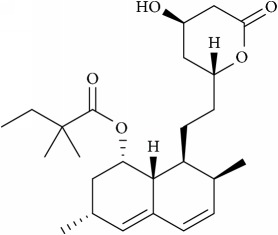	(i) Down-regulates PI3K/Akt/Caspase-3 signaling and Fas translocation (ii) Modulate raft assembly	[22]

(4) Resveratrol 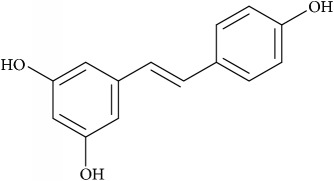	(i) Redistributes death receptor Fas in membrane rafts	[23]

(5) SB 204990 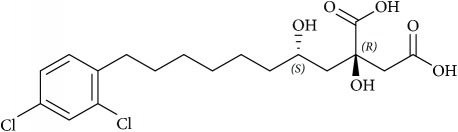	(i) ACLY inhibition (ii) Blocks FA and cholesterol synthesis	[24]

(6) Cerulenin 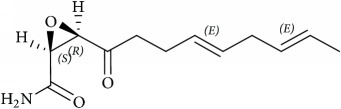	(i) Inhibitor of FA synthesis and reduces tumorigenesis	[25, 26]

(7) Etomoxir 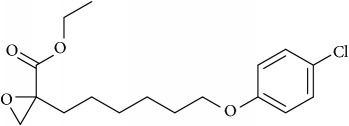	(i) Targets FA oxidation mediated via CPT1	[27]

(8) Perhexiline 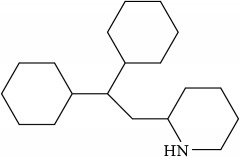	(i) Targets FA oxidation mediated via CPT1	[28]

(9) JZL 184 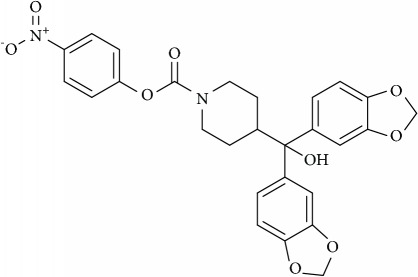	(i) Monoacylglycerol lipase (MAGL) inhibitor (ii) Inhibits mobilization of FAs from their reservoirs	[29]

(10) Deltarasin 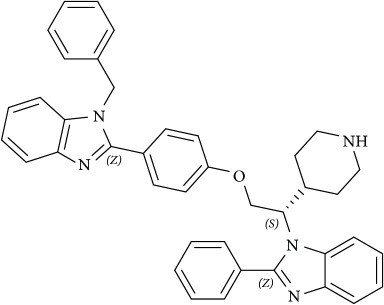	(i) Blocks PDE*δ* binding pocket (ii) Inhibits K-Ras lipoprotein tracking to plasma membrane	[30, 31]

(11) Vincristine 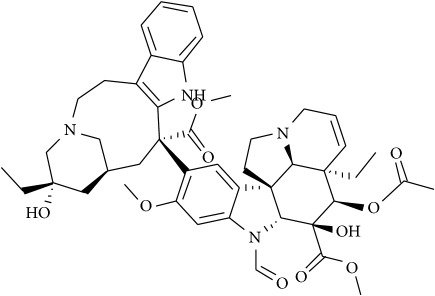	(i) Destabilizing agents (ii) Inhibits microtubule polymerization	[32, 33]

(12) Vinblastine 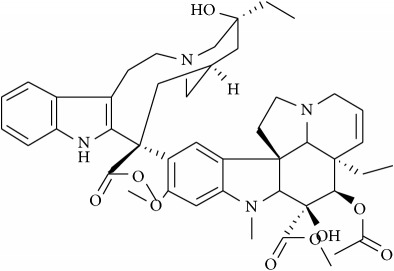	(i) Destabilizing agents (ii) Inhibits microtubule polymerization	[34, 35]

(13) Salirasib 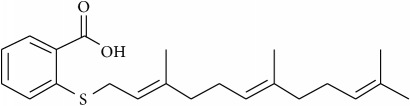	(i) Displaces farnesylated Ras from its binding pocket via competition (ii) Inhibits K-Ras binding to the plasma membrane	[36]
